# Letter from Prof. Bartlett

**Published:** 1847-01

**Authors:** 


*Letter from Prof. Bartlett.*—We publish the following letter from Prof. Bartlett, as the readiest and best mode of bringing the subjects before the profession of this State, and accomplishing the object which he desires. Our readers are aware that Prof. B. is the author of a work on Fevers, which has been deservedly much esteemed both at home and abroad, and is highly creditable to American medical literature. In responding to the demand for a new edition he is naturally desirous to make the work still more useful and complete. He is not satisfied with compiling from the works of other writers, but aims at increasing our stock of knowledge by the fruits of his own investigations, and by collecting the observations and experience of others. We shall lay the subject concerning which he asks information before the medical associations of this city and county, and in our next number endeavor to present the views which are entertained by the profession in this portion of the State. We would respectfully suggest that the same course be pursued in other portions, and we shall be happy to receive any communications which may be forwarded to us, and transmit them to Prof. B. either through the medium of our columns or otherwise. Respect for the praise worthy exertions of Prof. B. in behalf of the advancement of science, and native medical literature, should prompt all who feel an interest and pride in these matters to contribute whatever assistance may in their power.

Lexington, Kentucky; 10th Dec., 1846.

My dear Sir:—I am engaged in getting ready a second edition of my book on Fever; and there are some circumstances in relation to continued fever about which I am very desirous of obtaining further information. The history of continued fever, as it prevails in the New-England States, is very positively and fully ascertained; and it is now entirely certain, that it corresponds, in all respects, to the typhoid fever of the French. The same thing is true of the continued fever of this state; it is the common and most general fever of the country, and differs in no way from the usual fever of New-England. It is quite certain, further, that this same disease is extensively prevalent in Ohio. There is an opinion, pretty common in this region, that this form of fever is a new disease in this country; that it was not known formerly; and that it seems disposed to come in and take the place of malarious fevers. I am endeavoring to ascertain to what extent, if at all, this disease prevails through the Western and Southern States; and it would be a source of great gratification to me, if I could procue similar information in regard to New-York.

The characters of *Typhoid Fever*, as described by Louis, Dr. Nathan Smith, Dr. Jackson of Boston, and others, must be familiar to all your leading medical men throughout the state. *Is this the common form of fever in your state? If not the common form of fever, how frequently, and how extensively does it prevail? Is it less prevalent in those portions of the state that are subject to marsh fevers than in those where these latter diseases are not met with?*

Answers to these inquiries, communicated to me personally, or through the medium of your journal,—and any other information relating to this subject,—will be gratefully received. The matter is one of much scientific interest, and of great practical importance, and this must be my apology for addressing you. I have almost no personal acquaintance with the practitioners of your state, and I know of no other means of reaching them, than through the agency of your journals. I wish, if possible, to get some replies to my questions, between the present time and the first of March. I have the honor to remain, very respectfully, your friend,

ELISHA BARTLETT.

